# An Enjoyable Affair

**Published:** 1888-11

**Authors:** 


### AN ENJOYABLE AFFAIR.

By invitation we were present at a banquet given by the Odontological Society of New York, in honor of Dr. J. B. Davenport, of Paris, on the eve of his departure for home. Twenty-one covers were laid at Martinelli's, and a very enjoyable time was had. Dr. Davenport, although a young man, has already won for himself a name among the original investigators in dental science. The high character of his work was the theme of the evening. It was pleasant to see the older men willing to accord honor where honor was due, without that air of "pretty well done for a young man," that sometimes creep into grudgingly given praise. Encourage the young men, we say; give them a chance and a helping hand, if need be. Better be too lavish with praise than to stint it. We all like to be appreciated. Nothing serves as a stimulant to higher attainments more than to know that if there is not money, there is something better, viz., the satisfaction of having attained something which cannot be purchased with money, and to the possession of which there is no royal road. There were present, beside Dr. J. F. Davenport, as guests of the Society, a brother, Mr. D. T. Davenport, of Passedena, California, and an uncle, Dr. A. F. Davenport, of North Adams, Mass., Dr. J. F. D.'s former preceptor; and ye editor. The members present were:

| | |
|---|---|
| Drs. W. H. Dwinelle, *Chairman.* | William Jarvie, |
| Frank Abbott, | C. A. Woodward, |
| J. Morgan Howe, | C. F. Ives, |
| Benjamin Lord, | A. H. Brockway, |
| S. F. Howland, | F. A. Remington, |
| Z. T. Sailer, | W. A. Bronson, |
| J. Bond Littig, | George W. Weld, |
| S. G. Perry, | W. H. Atkinson, |
| H. G. Mirrick, | S. E. Davenport. |

One thing that impressed us was that the *International Dental Journal Company* was largely represented around the board, and we were farther rejoiced to obtain, during the evening, a promise from Dr. Davenport to act as a foreign correspondent for the *Journal* in Paris. We shall look for some valuable and interesting contributions from his pen. Word has been received of the doctor's safe arrival in Paris.

